# The c-Met Inhibitor MSC2156119J Effectively Inhibits Tumor Growth in Liver Cancer Models

**DOI:** 10.3390/cancers6031736

**Published:** 2014-08-19

**Authors:** Friedhelm Bladt, Manja Friese-Hamim, Christian Ihling, Claudia Wilm, Andree Blaukat

**Affiliations:** EMD Serono, and Merck Serono Research and Development, Merck KGaA, Darmstadt 64293, Germany; E-Mails: Manja.Friese-Hamim@merckgroup.com (M.F.H.); Christian.Ihling@merckgroup.com (C.I.); Claudia.Wilm@merckgroup.com (C.W.); Andree.Blaukat@merckgroup.com (A.B.)

**Keywords:** c-Met, hepatocellular carcinoma, hepatocyte growth factor, MSC2156119J, receptor tyrosine kinase, sorafenib, xenograft models

## Abstract

The mesenchymal-epithelial transition factor (c-Met) is a receptor tyrosine kinase with hepatocyte growth factor (HGF) as its only high-affinity ligand. Aberrant activation of c-Met is associated with many human malignancies, including hepatocellular carcinoma (HCC). We investigated the *in vivo* antitumor and antimetastatic efficacy of the c-Met inhibitor MSC2156119J (EMD 1214063) in patient-derived tumor explants. BALB/c nude mice were inoculated with MHCC97H cells or with tumor fragments of 10 patient-derived primary liver cancer explants selected according to c-Met/HGF expression levels. MSC2156119J (10, 30, and 100 mg/kg) and sorafenib (50 mg/kg) were administered orally as single-agent treatment or in combination, with vehicle as control. Tumor response, metastases formation, and alpha fetoprotein (AFP) levels were measured. MSC2156119J inhibited tumor growth and induced complete regression in mice bearing subcutaneous and orthotopic MHCC97H tumors. AFP levels were undetectable after 5 weeks of MSC2156119J treatment, and the number of metastatic lung foci was reduced. Primary liver explant models with strong c-Met/HGF activation showed increased responsiveness to MSC2156119J, with MSC2156119J showing similar or superior activity to sorafenib. Tumors characterized by low c-Met expression were less sensitive to MSC2156119J. MSC2156119J was better tolerated than sorafenib, and combination therapy did not improve efficacy. These findings indicate that selective c-Met/HGF inhibition with MSC2156119J is associated with marked regression of c-Met high-expressing tumors, supporting its clinical development as an antitumor treatment for HCC patients with active c-Met signaling.

## 1. Introduction

The mesenchymal-epithelial transition factor (c-Met) is a receptor tyrosine kinase with hepatocyte growth factor (HGF) as its only known high-affinity ligand. During embryonic development, c-Met controls morphogenesis, invasiveness, and migration of precursor cells. In adult life, the protein is typically expressed at low levels in a range of tissues, predominantly involved in tissue repair, and activated by pathologic stimulation. More specifically, c-Met is essential in liver development and regeneration. In conditional c-Met knockout mice, liver repair is delayed or absent after hepatectomy or chemically induced liver injury [1]. In contrast, overexpression of HGF has been shown to increase liver regeneration and to cause significant liver enlargement after partial hepatectomy in mice [2]. However, c-Met expression is deregulated in many human malignancies, including hepatocellular carcinoma (HCC) [3]. In the cancer setting, c-Met/HGF mediates cellular proliferation, tumor invasion, and metastasis [4]. The underlying biologic mechanisms for the tumorigenicity of c-Met appear to involve the establishment of c-Met/HGF autocrine loops, overexpression of c-Met or HGF, and kinase-activating mutations in the c-Met gene [5]. Overexpression of c-Met alone has been demonstrated to be sufficient for developing HCC in Met-transgenic mice [6,7]. In addition, high expression of c-Met has been observed in more than 80% of patients with HCC where it is correlated with poor progression-free survival and where it may be a predictor for sensitivity to agents such as the tyrosine kinase inhibitor sorafenib [8].

Although systemic treatment with sorafenib is the recommended treatment in advanced HCC, any survival benefit is limited, and novel tumor targets such as c-Met are warranted in this setting [9]. The use of c-Met inhibitors as a potentially viable treatment is supported by preclinical data showing that c-Met inhibition suppresses the growth of c-Met-positive HCC tumor cells [4,10]. The highly selective, high-potency, ATP-competitive c-Met inhibitor MSC2156119J has been shown to efficiently inhibit c-Met phosphorylation and downstream signaling *in vivo*, and to induce the regression of established tumors in xenograft models [11]. Recent studies indicate that these patient-derived tumor models may be highly predictive of patients’ response to therapy in the clinical setting [12,13]. In this study, tumor explants of c-Met-positive HCC patients were, therefore, grafted onto immunocompromised mice to evaluate the tolerability and the antitumor and antimetastatic activity of MSC2156119J compared to sorafenib. In addition, the effects of MSC2156119J in combination with sorafenib were evaluated.

## 2. Results and Discussion

### 2.1. Efficacy of MSC2156119J as Monotherapy or in Combination with Sorafenib or Rapamycin in MHCC97H Xenografts

#### Effect of MSC2156119J on Primary Tumors, Circulating AFP Levels, and Metastases Formation

**Figure 1:**
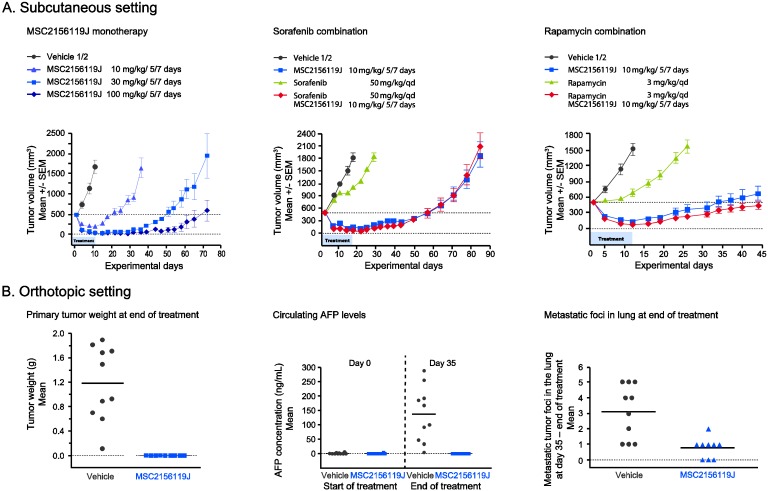
Efficacy of MSC2156119J in a patient-derived HCC xenograft model. (**A**) MHCC97H, subcutaneous setting; (**B**) MHCC97H, orthotopic setting. Treatment of MSC2156119J (10 mg/kg/5/7 days) started one week after tumor fragment implantation.

To investigate the effect of MSC2156119J as monotherapy or in combination with sorafenib or rapamycin on tumor viability *in vivo*, the patient-derived HCC cell line MHCC97H was used in a mouse xenograft model. MSC2156119J inhibited growth and induced regression of tumors grown either subcutaneously (Figure 1A) or orthotopically (Figure 1B).

In the subcutaneous setting, MSC2156119J displayed efficacy in a dose-dependent manner as a single agent (Figure 1A, first panel). During treatment, rapamycin displayed a moderate effect on tumor growth, while sorafenib was inactive (Figure 1A, second and third panel). The combination of a suboptimal MSC2156119J dose with sorafenib or rapamycin did not show superior efficacy of MSC2156119J on tumor viability. MSC2156119J also highly effectively inhibited the growth of orthotopic engrafted MHCC97H tumors. Treatment with 100 mg/kg of MSC2156119J was started 7 days after tumor fragment implantation. After 5 weeks of treatment, mice were sacrificed and a series of parameters were assessed, including serum AFP levels, whole liver weight, primary (intrahepatic) tumor size and weight. The intrahepatic tumor size and weight were significantly lower in MSC2156119J treated mice compared to the control group (*p* < 0.001) (Figure 1B, first panel). In 2 out of 9 mice no tumors were detectable at end of treatment. For the remaining mice, detectable tumor volumes at end of treatment were clearly below the size of tumor fragments which were initially inoculated in mice. Circulating AFP levels at endpoint were readily detectable in mice bearing orthotopic MHCC97H tumors in the control group, whereas AFP levels were undetectable after treatment with MSC2156119J (*p* < 0.001) due to inhibition of primary tumor growth (Figure 1B, second panel). Notably, liver engrafted MHCC97H tumors frequently metastasize to the lung. In the MHCC97H xenograft model, metastases formation was evaluated based on the number of mice with lung metastases and the number of metastatic foci. Compared to the control group, fewer animals developed lung metastases in the treated group (10 out of 10 *vs.* six out of nine, respectively). In addition, MSC2156119J treatment reduced the number of metastatic foci in the lungs of mice bearing orthotopic MHCC97H tumors, compared to the control group (*p* < 0.01; Figure 1B, third panel).

### 2.2. Efficacy of MSC2156119J and Sorafenib in HuPrime Primary Explant Xenograft Models

#### 2.2.1. Kinetics of Tumor Growth after MSC2156119J and Sorafenib Treatment

**Figure 2:**
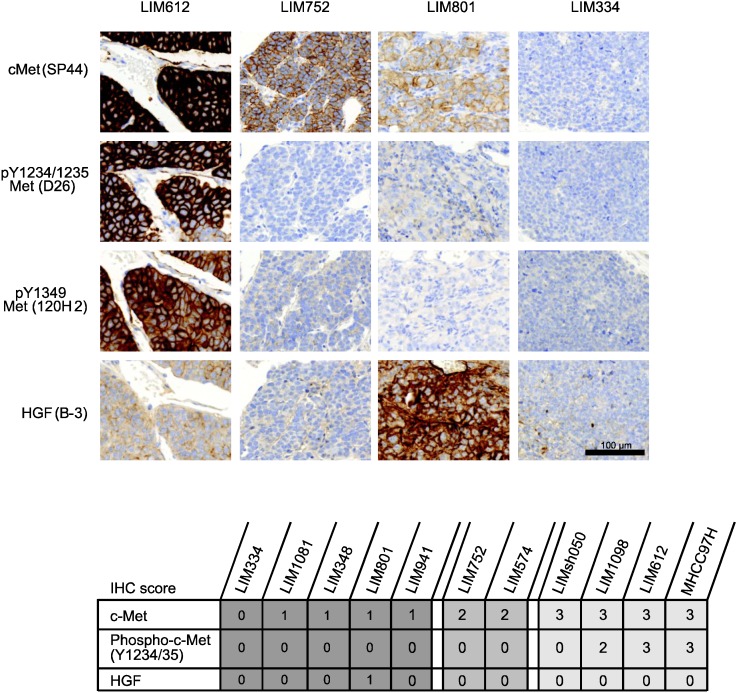
Expression of c-Met, HGF, and phospho-c-Met in patient-derived HCC explants.

The antitumor activity of MSC2156119J was further evaluated in HuPrime primary patient-derived human liver cancer explant xenograft models (see Supplementary Table S1), which were classified by immunohistochemistry (IHC) according to low (IHC score = 0–1), intermediate (IHC score = 2), or high (IHC score = 3) c-Met expression levels (Figure 2).

**Figure 3:**
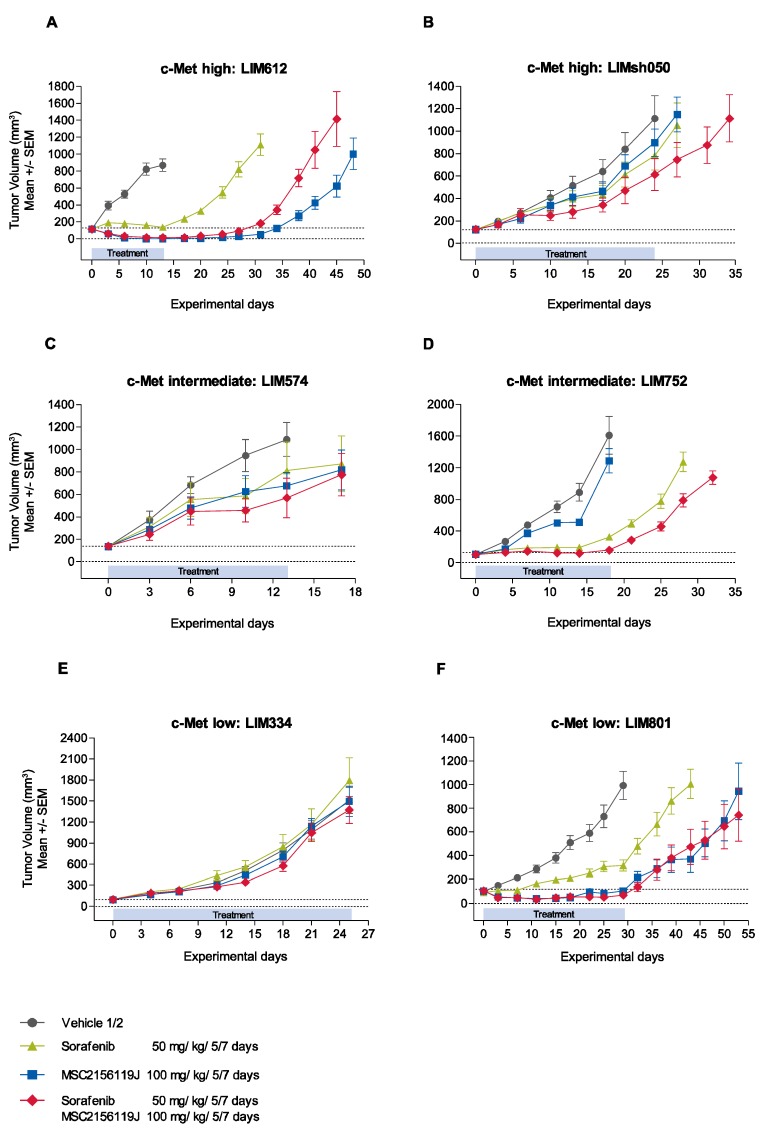
Representative examples of *in vivo* response kinetics of patient-derived HCC xenografts to MSC2156119J and sorafenib.

The kinetics of tumor growth in representative c-Met low, intermediate, and high expressing xenograft models are depicted in Figure 3. The high-c-Met-expressing model LIM612 showed significant antitumor activity in all three treatment groups (Figure 3A). MSC2156119J monotherapy induced complete tumor regression on day 13 while sorafenib induced tumor stasis. MSC2156119J in combination with sorafenib did not result in significantly enhanced antitumor activity compared with MSC2156119J as a single agent (*p* > 0.05). MSC2156119J monotherapy as well as sorafenib monotherapy were shown to be inactive in the high-c-Met-expressing model LIMsh050 (Figure 3B). However, MSC2156119J and sorafenib combination therapy enhanced the antitumor activity (median TV change 245%) compared with the vehicle group (median TV change 650%) on day 24, but tumors progressed under treatment.

For the intermediate c-Met-expressing models LIM574 and LIM752, no antitumor activity was observed after MSC2156119J monotherapy treatment (Figure 3C,D). Sorafenib monotherapy activity was observed in the LIM752 model resulting in a significant tumor growth inhibition (median TV change of 168%) compared to the vehicle control (median TV change of 1435%; *p* < 0.0001), but tumors still progressed under treatment. Combined treatment of MSC2156119J and sorafenib led to tumor stasis in the LIM752 model with a median TV change of 45% on day 18 compared with the vehicle group (*p* < 0.0001).

In the LIM334 model no c-Met levels could be detected, and treatment of tumors with either MSC2156119J or sorafenib monotherapy, or combination therapy resulted in tumor progression without any significant differences between the control and the three treatment groups (Figure 3E). In the low-c-Met-expressing LIM801 model, single-agent MSC2156119J showed strong efficacy leading to tumor stasis, with a significant difference in tumor size compared to the control group on day 29 (*p* < 0.0001; Figure 3F). Sorafenib monotherapy was less active than MSC2156119J monotherapy, and the addition of sorafenib to MSC2156119J did not enhance the activity of MSC2156119J.

In addition, AFP plasma levels were measured in these xenograft models at the start and at the end of treatment. AFP levels were detectable in five of 10 models used. Similar to the study in MHCC97H tumors, the change of AFP levels upon treatment correlated positively with the observed efficacy in the different treatment groups (see Supplementary Table S2).

#### 2.2.2. Treatment Tolerability

Treatment with MSC2156119J monotherapy was well tolerated in all models and no significant body weight loss was observed (representative cases are shown in Supplementary Figure S1). In contrast, sorafenib monotherapy administered at the maximum tolerated dose was associated with a body weight loss up to 10%. Similar body weight loss was observed in the MSC2156119J and sorafenib combination therapy.

#### 2.2.3. Tumor Response According to c-Met Expression Status

**Figure 4:**
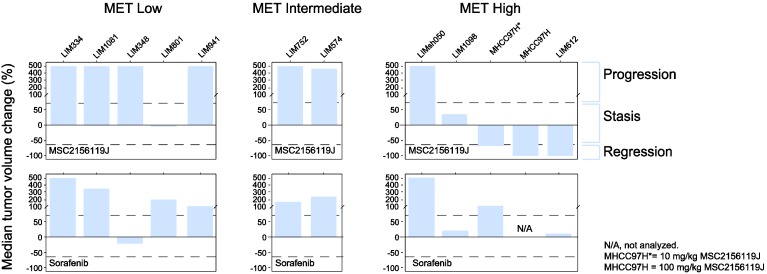
Response of patient-derived HCC xenografts to MSC2156119J and sorafenib.

Of the 11 xenograft models used in this study, five models expressed low levels of c-Met, two models expressed intermediate c-Met levels, and four models showed high c-Met levels (Figure 2). None of these models have detectable human HGF levels except LIM801, which co-expresses HGF and low levels of c-Met (autocrine model). Figure 4 shows that three of four (75%) of the c-Met-high-expressing patient-derived xenografts responded to MSC2156119J monotherapy. In two models tumor regression was observed, and tumor stasis was seen in one primary explant. In contrast, no response was observed in c-Met-intermediate models and only one c-Met-low-expressing xenograft, the autocrine model LIM801, displayed a response to MSC2156119J monotherapy (tumor stasis). Sorafenib monotherapy induced tumor stasis in two of four (50%) of c-Met-high-expressing xenografts, while no tumor regression was observed.

The observation that the c-Met-low-expressing xenograft model LIM801 responded to MSC2156119J monotherapy could be explained by the fact that this is an autocrine model, co-expressing HGF and c-Met (Figure 2). To better understand the molecular basis for the activity of MSC2156119J, phospho-c-Met levels (*i.e.*, c-Met-activating status) were determined. Table 1 and Figure 2 show that only barely detectable phospho-c-Met levels (IHC score = 0) could be observed for the autocrine, low-c-Met-expressing model LIM801. No phospho-c-Met levels were detected in the other c-Met-low-expressing models, which all showed tumor progression.The three c-Met-high HCC xenografts (LIM1098, MHCC97H, and LIM612) that responded to MSC2156119J (two tumor regressions, one tumor stasis) expressed high levels of phospho-c-Met levels. In contrast, c-Met phosphorylation was undetectable in the fourth high-c-Met-expressing model LIMsh050 and the two intermediate-c-Met-expressing models (LIM752 and LIM574) that were not sensitive toward MSC2156119J inhibition.

### 2.3. Discussion

In this study, we evaluated the antitumor effects of the c-Met inhibitor MSC2156119J in the treatment of 11 patient-derived tumor explants grafted onto immunocompromised mice in a subcutaneous and orthotopic setting. Our findings provide evidence that MSC2156119J inhibits the growth of a subset of HCC tumors characterized by high c-Met expression levels and suggest that this compound may be a promising candidate for cancer therapy. Specifically, the data show that MSC2156119J effectively inhibited growth and induced regression of tumors with high c-Met protein levels, with less activity apparent in tumors expressing intermediate or low detectable c-Met levels. Of special interest was the observation that MSC2156119J also reduced the number of metastatic foci in the lung. HGF/c-Met signaling is known to be associated with metastasis by mediating a complex program known as invasive growth [14,15]. We can, however, not rule out that the effect of MSC2156119J treatment on the primary tumors partially contribute to the observed anti-metastatic effect in the orthotopic engrafted HCC model. MSC2156119J showed similar or superior efficacy to sorafenib in tumor models characterized by a high c-Met score. While sorafenib treatment induced up to 10% body weight loss, MSC2156119J was well tolerated in mice and was not associated with loss of body weight even at the highest dose as single agent (100 mg/kg). Administration of MSC2156119J together with sorafenib did not enhance efficacy compared with the respective monotherapies. The combination treatment was shown to increase toxicity of MSC2156119J, similar to sorafenib monotherapy. Our data additionally demonstrate that treatment with MSC2156119J was associated with ablation of AFP in conjunction with tumor regression. In line with our results, high levels of AFP, a clinicopathologic marker of HCC, have been indirectly correlated with other prognostic markers, including c-Met, in tissue samples from patients with HCC [8,16].

**Table 1:** Efficacy of MSC2156119J and c-Met activation status in patient-derived HCC xenograft models with low (IHC score = 0–1), intermediate (IHC score = 2), and high (IHC score = 3) c-Met levels.

	Efficacy		
	Regression	Stasis	Progression
**Activated c-Met receptor** (phospho-c-Met positive)			
c-Met low	0/0	0/0	0/0
c-Met intermediate	0/0	0/0	0/0
c-Met high	2/3	1/3	0/3
**Nonactivated c-Met receptor** (phospho-c-Met negative)			
c-Met low	0/5	1/5	4/5
c-Met intermediate	0/2	0/2	2/2
c-Met high	0/1	0/1	1/1

Several preclinical studies have demonstrated that c-Met is able to function as an oncogenic driver for the initiation and progression of HCC. Deregulation of c-Met activation in cancer, leading to an aggressive tumor phenotype, poor prognosis, and resistance to targeted therapy, can be caused by various mechanisms, such as protein overexpression, gene amplification, autocrine (*i.e.*, c-Met and HGF co-expression) and paracrine activation (*i.e.*, tumor-mediated c-Met expression, together with tumor microenvironment-regulated HGF expression), presence of activating point mutations, or downregulation of c-Met-targeting miRNAs [17]. The ligand of c-Met was discovered as a growth factor for liver (HGF). The role of HGF/c-Met in liver development and maintenance highlights the potential role HGF/c-Met can play in liver cancer development. It has been described that HGF and c-Met-null mice have an impaired liver development [18,19], and downregulation or inhibition of the receptor interferes with both cell growth and cell invasiveness *in vitro* and *in vivo* [20,21]. Autocrine activation of c-Met was shown to drive the metastatic process in mice by leading to the development of spontaneous metastases and to predict sensitivity to c-Met inhibitors in *in vivo* glioblastoma models [22,23,24].

Based on these studies and increasing preclinical evidence, a number of c-Met/HGF inhibitors are currently under study in several clinical trial phases. Tivantinib, initially reported to be a selective, non-ATP-competitive c-Met inhibitor [25], is now being tested in a Phase II trial (METIV) on the basis of the results obtained in a Phase II trial where the efficacy of the drug was tested as second-line treatment in patients with advanced-stage HCC and Child-Pugh A cirrhosis [26]. Time to progression (TTP) was longer for patients who received tivantinib *vs.* placebo (1.6 and 1.4 months, respectively; *p* = 0.04) and high c-Met expression correlated with a more positive outcome (median TTP of 2.7 months in tivantinib arm *vs.* 1.4 months in the placebo arm). Two studies, however, have provided evidence against the hypothesized c-Met–targeting mechanism of action of tivantinib [27,28]. The results suggest that tivantinib exhibits its antitumor activity by targeting microtubules, independent of c-Met status.

The present data show that the antitumor activity of MSC2156119J treatment in multiple human liver cancer explant models is related to c-Met expression. Marked tumor growth inhibition and regression were associated with high protein levels of c-Met, with explants that expressed low levels of c-Met tumors not as sensitive to MSC2156119J. Of the four c-Met-high-expressing explants, one model failed to respond to MSC2156119J. Analysis of the activation of the c-Met receptor showed that all tumors with detectable phospho-c-Met levels responded to MSC2156119J treatment. In the model that failed to respond, no detectable c-Met receptor activation was found. In earlier studies, we have shown that c-Met autophosphorylation is inhibited effectively in a dose-dependent manner in the absence of alternations in c-Met expression levels. In all models with detectable phospho-c-Met baseline levels tested so far, phospho-c-Met is inhibited by MSC2156119J. PK/PD studies revealed that at doses of 10 mg/kg or more, MSC2156119J resulted in more than 90% inhibition of c-Met phosphorylation for a period of at least 72 h [11].

In patients, HGF produced by stromal cells in a paracrine manner may have an important stimulatory role for c-Met, in contrast to murine HGF, which is unable to activate the human c-Met receptor on the human xenografts. Therefore, only HGF-independent or autocrine models can benefit from c-Met inhibition in mice. IHC experiments were unable to detect phosphorylated c-Met, demonstrating that the overexpression of c-Met in these tumors was not sufficient to activate the c-Met receptor. This finding implies that the c-Met pathway was not the tumor “driver”, or that these tumors have been driven by a paracrine HGF mechanism in the patient that cannot be reproduced in the xenograft setting. The critical role of HGF in further activating overexpressed c-Met receptors is supported by the finding that inhibitors blocking the interaction of HGF and c-Met show some activity in c-Met-overexpressing/amplified patients [29]. The murine models used in this paper may therefore cause an underestimation of the efficacy of MSC2156119J in c-Met high-expressing tumors.

In addition, the observation that the low c-Met-expressing LIM801 xenograft model did display sensitivity to MSC2156119J could be explained by co-expression of c-Met and HGF in these cells. Studies revealed that autocrine models are characterized by low levels of c-Met and clearly detectable HGF levels [11]. Phosphorylated c-Met receptors are often not detectable due to insufficient sensitivity of the applied assays, such as IHC and western blot. Only highly sensitive Luminex assays are able to detect the very low levels of phospho-c-Met present in these autocrine tumors. In current clinical practice, selection of patients occurs mainly on the basis of c-Met overexpression or amplification. An established assay to identify patients with low levels of c-Met where the c-Met receptors are activated by paracrine/autocrine HGF would allow the identification of additional patients who might benefit from c-Met-targeting therapies. Since high HGF facilitates activation of c-Met even at lower levels by paracrine/autocrine feedback, the determination of HGF levels in these patients is warranted.

An additional observation that supports the detection of HGF levels in HCC patients comes from Xie *et al.* who demonstrated that overexpression of human HGF in mice can induce the overproduction of hepatitis B virus surface antigen (HBsAg) [30]. Chronic hepatitis B virus (HBV) infection is a well-known cause of HCC, and although the underlying mechanism is yet unclear, *in vivo* studies have shown that overexpression of HBsAg is sufficient to cause malignant transformation of the liver [31]. In addition, overexpression of HGF produced a more aggressive HCC phenotype in these mice [30]. It is therefore possible that c-Met activation contributes to HBV-induced HCC. Interestingly, in our study, three out of four xenografts that responded to monotherapy MSC2156119J (MHCC97H and LIM612 and the low c-Met-expressing LIM801) are highly positive for HBV.

Moreover, new data have emerged recently on the invasive capacity of c-Met/HGF-expressing tumor cells that established an epigenetic link between mixed-lineage leukemia (MLL) and the HGF-Met signaling pathway, which might aid in the stratification of patients for Met-targeting therapies [32]. In a subclass of HCC patients with high c-Met/HGF expression, the MLL coactivator has been shown to be induced, triggering a signaling cascade that conferred invasive tumor growth and seeding distant metastasis [33]. These patients might benefit from targeted therapy with MLL, taspase-1, or c-Met inhibitors, to potentially improve their survival.

Taken together, the data presented supports a critical role for c-Met in HCC. Patients with activated c-Met receptor, determined by c-Met overexpression or autocrine HGF production, are sensitive to c-Met inhibition and therefore more likely to show a clinical response to c-Met targeting agents.

## 3. Experimental

### 3.1. Compounds

MSC2156119J (EMD 1214063) was originally developed and synthesized at Merck Serono (Darmstadt, Germany). Sorafenib and rapamycin were provided by CrownBio (Beijing, China) and were ordered from Nanjing Ange Pharmaceutical Co., Ltd. (Nanjing, China), and Wyeth (Madison, NY, USA), respectively.

### 3.2. Animals and Housing

BALB/c nude male mice aged six-eight weeks and weighing 18–22 g (xenograft model), and BALB/c nude female mice aged six-eight weeks and weighing 18–22 g (HuPrime^®^ model) were housed in micro-isolation cages in groups of four animals in laminar flow rooms at a constant temperature of 20–26 °C and humidity of 30%–70%, with a 12-h light and 12-h dark cycle. Animals were fed an *ad libitum* diet of irradiated and sterilized dry food and sterile drinking water. Bedding was sterilized before use. An acclimation period of one week was allowed between animal receipt from the Shanghai Laboratory Animal Center (Shanghai, China) and tumor inoculation experiments, which were conducted at the Shanghai Liver Cancer Institute (Shanghai, China). All procedures relating to animal handling, care, and treatment were performed according to guidelines approved by the Institutional Animal Care and Use Committee of CrownBio, further in accordance with the Association for Assessment and Accreditation of Laboratory Animal Care.

### 3.3. MHCC97H Human HCC Xenograft Model

The patient-derived HCC cell line MHCC97H (Shanghai Liver Cancer Institute) is known for its cancer stem cell-like characteristics [4], spontaneous lung metastases in the orthotopic tumor model [34], co-expression of HGF and c-Met in an autocrine loop [35], and secretion of alpha-fetoprotein (AFP) [35]. When grown either subcutaneously or orthotopically, MHCC97H cells are highly sensitive to c-Met inhibition.

To analyze the antitumor activity of MSC2156119J, human MHCC97H tumor cells were cultured in DMEM supplemented with 10% fetal bovine serum at 37 °C in an atmosphere of 5% CO_2_ in air. MHCC97H cells (passage 3) were harvested in exponential growth phase and counted for tumor inoculation. The tumor cells were subcutaneously inoculated in male BALB/c nude mice. When the tumors reached approximately 1 cm in diameter, subcutaneous tumors were collected and cut into pieces of about 2–3 mm^3^ and inoculated into the left lobe of the liver of male BALB/c nude mice.

Mice were treated orally five days on/two days off with either vehicle combination (*n* = 10; 20% Solutol/80% 100 mM Na-acetate buffer, pH 5.5 [Merck Serono]), MSC2156119J (*n* = 10; 10, 30, or 100 mg/kg), sorafenib (*n* = 10; 50 mg/kg), or rapamycin (*n* = 10; 3 mg/kg) as single-agent treatment. In addition, MSC2156119J (10 mg/kg) was given as a combination treatment with sorafenib (*n* = 10, 50 mg/kg) or rapamycin (*n* = 10; 3 mg/kg). Treatment was started seven days after orthotopic implantation of tumor fragments and terminated after five weeks. Six weeks after orthotopic inoculation, mice were sacrificed and whole liver weight, and primary tumor size and weight were measured. Tumor size was measured in two dimensions using a caliper and the volume was expressed in mm^3^ using the formula V = 0.5 a × b^2^, where a and b are the long and short diameters of the tumor, respectively.

Immediately before the first dose of MSC2156119J and at study termination, blood samples were collected from all animals. Serum AFP levels were determined by ELISA (Zhengzhou Biocell Inc., Henan, China) using a Synergy 2 Multi-Mode Microplate Reader (BioTek, Beijing, China) and analyzed by Gen5 Data Analysis software (BioTek).

At necropsy, the lungs were inflated with 10 mL of 10% buffered formalin through the trachea and both the left and right lungs of each animal were collected, immediately fixed in 10% buffered formalin, and embedded in paraffin. Paraffin blocks were cut into serial sections and stained with hemotoxylin and eosin. For each animal, three sections cut at 6-μm intervals were examined for incidence of lung metastases and the number of metastatic foci.

### 3.4. HuPrime Model with Human HCC Explants

In addition to the patient-derived HCC xenograft model, 10 primary human liver cancer explant models (HuPrime tumor grafts, CrownBio) were selected on the basis of their c-Met expression levels: low expression models were LIM334, LIM1081, LIM348, LIM801, LIM941; intermediate expression models consisted of LIM752 and LIM574; and high expression models included, LIMsh050, LIM1098, and LIM612. The pathology diagnosis of these patient-derived HCC models is shown in Supplementary Table S1.

Tumor samples were derived from 10 HCC patients, and were thereafter maintained subcutaneously in nude mice. When tumors reached 500–700 mm^3^ in volume, they were harvested for inoculation. Tumor fragments of 3 × 3 × 3 mm were implanted subcutaneously in the right rear flank of female BALB/c nude mice as described by the HuPrime tumor inoculation standard operating procedure (SOP-HP-001, CrownBio, Beijing, China).

A five-days-on/two-days-off treatment schedule (up to five cycles) with vehicle (*n* = 12), MSC2156119J (*n* = 12; 100 mg/kg, 5 μL/g), sorafenib (*n* = 12; 50 mg/kg), or the combination of MSC2156119J and sorafenib (*n* = 12) was initiated when the average tumor size reached approximately 100 mm^3^. Blood samples were taken before start of the treatment and end of the study for AFP determination.

The major efficacy endpoint was to evaluate the tumor growth rates. The evaluation of the antitumor activity was based on RECIST-like tumor response criteria using tumor volume (TV), as described by Therasse *et al.* [36] and based on median percentage of TV change at the end of treatment compared to the start of treatment. The activity was calculated as TV change in %: (TV end of treatment-TV start of treatment/TV start of treatment) × 100. Tumor progression was defined as a tumor volume change >73% at the end of the treatment compared to the TV at start of treatment. Tumor stasis has been defined as a tumor volume change of >−65% and ≤73% at the end of the treatment compared to the TV at start of treatment. Tumor regression was defined as a tumor volume change of ≤−65% at the end of the treatment compared to the TV at the start of treatment. Nonpalpable tumors or tumor volume (or mean tumor volume) <20 mm^3^ at end of treatment have been defined as complete tumor regression.

### 3.5. Immunohistochemistry

Treatment-naive patient-derived HCC explant models were immunostained for c-Met (rabbit IgG clone SP44, Ventana Medical Systems, Tucson, AZ, USA), phospho (Y1234/35)-Met (rabbit IgG clone D26, Cell Signaling Technology, Danvers, MA, USA), phospho (Y1349)-Met (rabbit IgG clone 130H2, Cell Signaling Technology), and HGF (mouse IgG clone B-3, Santa Cruz Biotechnology, Dallas, TX, USA) on formalin-fixed, paraffin-embedded sections. IHC was performed using the Discovery XT staining instrument (Ventana Medical Systems), applying the OmniMap anti-Rb HRP Detection Kit (Ventana Medical Systems), according to the manufacturer’s instructions. For the detection of HGF with the mouse IgG clone B-3, a rabbit monoclonal antimouse IgG antibody was used as secondary antibody followed by OmniMap anti-Rb HRP. Staining protocols were optimized using c-Met, phospho-Met and HGF-positive (high and medium expressing) and -negative cell lines. Sections were counterstained with hematoxylin.

The IHC stains of c-Met, phospho-Met, and HGF were scored semiquantitatively by a surgical pathologist using the scoring system described for c-Met [29]. Positive c-Met staining was defined as high (IHC score = 3; ≥50% tumor cells with membrane and/or cytoplasmic staining with strong intensity) or intermediate (IHC score = 2; ≥50% tumor cells with membrane and/or cytoplasmic staining with moderate or higher intensity but <50% tumor cells with strong intensity). Negative (low) c-Met staining was defined as IHC score 1 (≥50% tumor cells with membrane and/or cytoplasmic staining with weak or higher intensity but <50% tumor cells with moderate or higher intensity), or 0 (<50% tumor cells with membrane and/or cytoplasmic staining [could be combination of any staining intensities]).

### 3.6. Statistical Analyses

The mean between-group differences for body weight, liver weight, and tumor size and weight were assessed using either the Student’s t-test (human MHCC97H HCC cell line xenograft model) or two-way RM- ANOVA (HuPrime models). Contingency table analysis and chi-square tests were used to determine the relationship between treatment and lung metastasis. The standard error of the mean is reported along with mean values. A *p* value of ≤0.05 was considered to be statistically significant.

## 4. Conclusions

In conclusion, these preclinical data show that selective c-Met/HGF inhibition with MSC2156119J is associated with marked regression of c-Met/HGF-expressing human tumors in mouse xenograft models. Superior efficacy of MSC2156119J compared to sorafenib was observed in c-Met high-expressing tumors, because selection of c-Met high-expressing HCC enriches for tumors that are driven by c-Met. Our data support the clinical development of MSC2156119J as an antitumor treatment for HCC patients with active c-Met signaling, and the enrichment strategy should enable selection for patients with the highest likelihood of response to MSC2156119J.

## Supplementary Files

Supplementary File 1
