# Supplementary material for: The c-Met Inhibitor MSC2156119J Effectively Inhibits Tumor Growth in Liver Cancer Models
Source: Cancers (Basel). 2014 Aug 19;6(3):1736–52. doi: 10.3390/cancers6031736 (PMC4190565; doi:10.3390/cancers6031736)
Supplement: Supplementary File 1 [file cancers-06-01736-s001.pdf]

Supplementary Information

Table S1. Pathology diagnosis of the patient-derived HCC models used in this study.

Model Name	Pathology Diagnosis (Provided by CrownBio)	Other
LIMsh050	Hepatocellular carcinoma from middle lobe of liver, grade IV, G4-S4, cancer emboli were observed in part of vessels, nodular cirrhosis presented in surrounding liver tissues, fatty degeneration (about 10%–15%) was observed.	HBV(−); HCV(−)
LIM334	Nodular cirrhosis with hepatocellular carcinoma, chronic cholecystitis.	HBV(+++); HCV(−)
LIM348	Postnecrotic cirrhosis with combined cell type carcinoma; postnecrotic cirrhosis with mixed carcinoma of liver.	Cachexia; HBV(−); HCV(−)
LIM574	Hepatocellular carcinoma.	HBV(±); HCV(−)
LIM612	Postnecrotic cirrhosis with hepatocellular carcinoma. Chronic cholecystitis.	Cachexia, HBV(+++); HCV(−)
LIM752	Hepatocellular carcinoma of right lobe of liver with massive necrosis, massive type, tumor mass: 14 cm × 12 cm × 10 cm. Chronic cholecystitis, cholelithiasis. IHC result: CK(++) CK7(−) CK18(++) CK19(++) CEA(−) AFP(−) Hepatocyte(−) Actin(−) Actin(SM)(−) CD34(+) F8(+).	HBV(−); HCV(−)
LIM801	Early hepatocirrhosis with hepatocarcinoma. Chronic cholecystitis. Cholelithiasis.	HBV(++); HCV(−)
LIM941	Hepatocirrhosis with hepatocarcinoma. Malignant cells invaded gall bladder wall.	Cachexia; HBV(±); HCV(−)
LIM1081	Hepatocellular carcinoma, diffuse in liver, cancer embolus in vessel. IHC results: AFP(<5% +), HEPA(100% +++), CD34(massive vessel), CK19(–), PDEC(10% +), P53(20% ++ − +++), EGFR(–), HER2(<5% +), KI-67(about 15% ++).	HBV(+++); HCV(−)
LIM1098	Hepatocellular carcinoma of right lobe, grade II, peripheral hepatic tissue G1S1.	Cachexia, HBV(−); HCV(−)
MHCC97H	Highly metastatic clone from parental HCC cell line LCI-D20, LCI-D20 model established from a highly metastatic patient-derived hepatocellular carcinoma.	[1]

Table S2. AFP plasma levels in xenograft models at the start and at end of treatment.

Liver Cancer Model	AFP Plasma Concentration (ng/mL)								
	Vehicle			Sorafenib			MSC2156119J		
	Before Start of Treatment	End of Treatment	Fold Change	Before Start of Treatment	End of Treatment	Fold Change	Before Start of Treatment	End of Treatment	Fold Change
**LIM801**	33	134	**4.1**	18	23	**1.3**	19	35	**1.8**
**LIM574**	7671	25638	**3.3**	5817	9017	**1.6**	5929	14922	**2.5**
**LIM752**	925	15995	**17.3**	1012	1629	**1.6**	847	16210	**19.1**
**LIMsh050**	4025	11944	**3.0**	4321	30815	**7.1**	4984	33916	**6.8**
**MHCC97H**	1	136	**136**	NA	NA	**NA**	24	6	**0.25**

No detectable AFP levels in model LIM334, LIM1081, LIM348, LIM1098. LIM941 and LIM612—subcutaneous tumor model; MHCC97H orthotopic tumor model.

Figure S1. Body weight change over time in response to MSC2156119J, sorafenib monotherapy, and combination treatment in three representative human explant xenograft models. Model LIM612 is a cachexia inducing model as seen by the body weight loss in vehicle group. Sorafenib treatment resulted in a similar body weight loss as observed in the vehicle group despite inhibiting tumor growth.

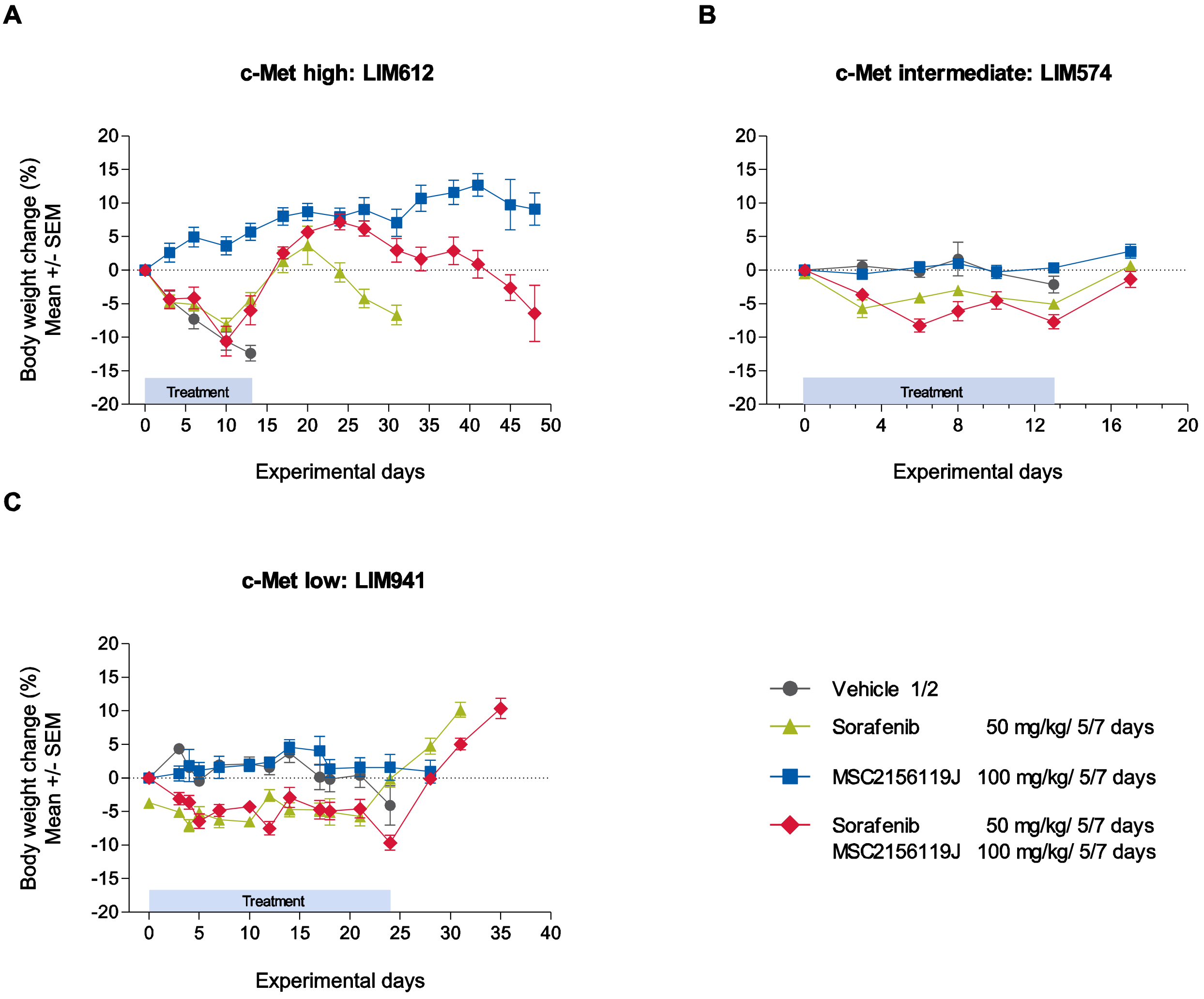

References

1. Sun, F.X.; Tang, Z.Y.; Lui, K.D.; Ye, S.L.; Xue, Q.; Gao, D.M.; Ma, Z.C. Establishment of a metastatic model of human hepatocellular carcinoma in nude mice via orthotopic implantation of histologically intact tissues. *Int. J. Cancer* **1996**, *66*, 239–243.

© 2014 by the authors; licensee MDPI, Basel, Switzerland. This article is an open access article distributed under the terms and conditions of the Creative Commons Attribution license (http://creativecommons.org/licenses/by/3.0/).
